# Physical and Psychological Effects of Head Treatment in the Supine Position Using Specialized Ayurveda-Based Techniques

**DOI:** 10.1089/acm.2015.0388

**Published:** 2016-07-01

**Authors:** Masako Murota, Yoko Iwawaki, Kazuo Uebaba, Yoko Yamamoto, Yukie Takishita, Kiyomi Harada, Akemi Shibata, Jin Narumoto, Kenji Fukui

**Affiliations:** ^1^ School of Nursing, Kyoto Prefectural University of Medicine, Kyoto, Japan.; ^2^ Department of Human Care, Teikyo Heisei University, Tokyo, Japan.; ^3^ Department of Psychiatry, Graduate School of Medicine, Kyoto Prefectural University of Medicine, Kyoto, Japan.

## Abstract

***Objective:*** To clarify the physical and psychological effects of head massage performed in the supine position using Ayurveda-based techniques (head treatment).

***Design:*** Twenty-four healthy female students were included in the study. Using a crossover study design, the same participants were enrolled in both the head treatment intervention group and control group. There was an interval of 1 week or more between measurements.

***Outcome measures:*** The physiologic indices measured included blood pressure and heart rate fluctuations (high frequency and low frequency/high frequency). The psychological markers measured included liveliness, depression, and boredom using the visual analogue scale method. State anxiety was measured using the State-Trait Anxiety Inventory method.

***Results:*** The parasympathetic nerve activity increased immediately after head treatment. Upon completion of head treatment, the parasympathetic nerve predominance tended to gradually ease. Head treatment boosted freshness and relieved anxiety.

***Conclusions:*** The results suggest that head treatment has a relaxing and refreshing effect and may be used to provide comfort.

## Introduction

A head treatment is a massage to the head, and it is considered to affect physical and psychological relaxation and to be refreshing. Researchers have examined the physical and psychological effects of head treatment and have reported that head treatment in the supine position is a nursing care technique useful for providing comfort.^1–3^

Use of this technique will enable nurses to provide relaxation to patients who are unable to maintain a sitting position. If the relaxing effects of head treatment in the supine position could be clarified, patients would be able to receive comfort through an intervention that can be individually adapted.

Therefore, the purpose of the present study was to clarify the physical and psychological effects of head treatment in the supine position by using a specialized Ayurveda-based technique, measured according to physiological and psychological indices.

## Materials and Methods

### Participants

The researchers recruited 24 healthy female students. This study was conducted with a crossover design in which the same participants were included in the intervention group that underwent a head treatment and the control group in which bed rest was maintained. These two treatments were administered with an interval of more than 1 week between the treatments. The study was conducted at K University training room from July to August 2012, with temperature and humidity controlled at 24°C and 60%, respectively. To ensure a certain skill level of the practitioner and that study results were due to the effect of head treatment, all massages were performed by a therapist from the educational institution of Indian head massage.

### Intervention procedure

The intervention is described in Table 1. Before the intervention, participants were asked to record psychological indices on a self-administered questionnaire survey. Thereafter, physiologic indices were measured. During the 7-minute treatment, the intervention group received head treatment and the control group rested in bed. Immediately after treatment, physiologic indices were measured. Thereafter, the participants rested for 15 minutes. Physiologic indices were measured again at 15 minutes after treatment. After evaluation, participants were asked to record psychologic indices on a self-administered questionnaire. The head treatment was performed by using the 7-minute head treatment program in the supine position (July 2012 edition, developed in collaboration with Yoko Miyazaki (Table 2).

**Table 1.** Intervention Procedure

			*Physiologic measures*			*Psychological indices*		
*Action*	*Time required (min)*	*Position*	*SBP/DBP*	*HF*	*LF/HF*	*VAS*	*STAI*	*Self-recorded questionnaire*
1. Enter room and change clothes								
2. [Before start] VAS STAI questionnaire	5	Sitting				○	○	○
3. Take each measurement	3		○					
4. [Intervention]	7							
Intervention group: head treatment								
Control group: bed rest in supine position				Continuously measured				
5. [Immediately after intervention] Take each measurement	15	Supine position	○					
6. Rest in supine position								
7. [30 min after intervention] Take each measurement	3		○					
8. VAS STAI questionnaire	5	sitting				○	○	○

SBP, systolic blood pressure; DBP, diastolic blood pressure; HF, high frequency; LF, low frequency; VAS, visual analog scale; STAI, State-Trait Anxiety Inventory; ○, data collection point.

**Table 2.** Head Treatment Procedure

*Head treatment procedure*	*Time required (s)*	*Related* marma *point*
1. Hold the head with both palms and all 5 fingers and rub the temporal region up and down.	20	*Utkshepa*
2. Gently rub all over the temples using a back and forth motion.	30	*Shankha*
3. Move the finger tips in a back and forth motion on both temporal regions.	15	*Utkshepa*
4. Massage the temporal regions, forehead, and back of the head with the fingers of both hands.	15	*Utkshepa, Adhipati, Simantakas*
5. Apply acupressure on the middle of the head from the hairline using front to back motion.	20	*Simantakas*
6. Press the thumbs on either side of the head approximately 3 cm from the middle using a front to back motion.	10	
7. Place the thumbs on both sides of the pressure points at the base of the neck and apply pressure so as to pull up the head.	20	*Krikatikas*
8. Press the right thumb on the pressure point at the base of the right side of the neck slightly to the middle while lifting the neck.	20	*Vidhuram, Krikatikas, Siramatrika*
9. Press the left thumb on the pressure point at the base of the left side of the neck slightly to the middle while lifting the neck.	20	
10. Place the thumbs on the depression of the back of the head and press on it so as to lift the neck.	20	
11. While shifting the body weight backward, pull the neck upward with both thumbs moving on both sides from the pressure points at the base of the neck toward the *naohu* point.	10	
12. Press the fingers on the trapezius muscle along its point of attachment with the shoulder blade.	20	*Ansa-phalak*
13. With the right arm raised above the shoulder, press the right thumb into the center of the trapezius muscle from the top.	20	*Ansa*
14. With the left arm raised above the shoulder, press the thumb into the center of the trapezius muscle from the top.		
15. With the palm cupped, gently and repeatedly tap both sides of the head, the back of the head, the shoulders, and the back to the deltoid muscle area.		*Utkshepa, Simantakas, Krikatikas, Siramatrika, Ansa, Ansa-phalak*
16. Rub from the head down to the shoulders and back.	10	
17. Rub the right arm in a downward motion, alternating both hands, then rub the left arm in a downward motion, alternating both hands.	20	

### Test items

Physiologic indices were blood pressure and heart rate fluctuation index. Blood pressure was measured by using a HEM-642 automated digital sphygmomanometer (Omron Healthcare Co., Ltd, Kyoto, Japan). To measure heart rate fluctuation index, a radiofrequency-electrocardiography wireless vital sensor (GMS Co., Ltd, Kyoto, Japan) was attached to the participants' chests to record electrocardiographic complexes. Frequency analysis was performed by using the maximum entropy method with MemCalc/Bonaly Light real-time analysis software (GMS Co., Ltd, Kyoto, Japan) for heart rate fluctuation based on the RR interval. In the frequency analysis, the ratio of low-frequency (LF) domain (0.04–0.15 Hz) to high-frequency (HF) domain (0.15–0.40 Hz) was calculated as mean/10 seconds. The HF domain is generally considered a true indicator of the cardiac vagus nerve function (parasympathetic nerve activity). The LF domain reflects the activities of both the cardiac vagus nerve and cardiovascular sympathetic nerve systems; thus, the LF/HF domain is generally considered as an indicator of cardiac sympathetic nerve activity.^4^

### Psychological indices

Liveliness, depression, and boredom were measured by using a visual analog scale (VAS) as three appropriate indices of relaxation. Anxiety was measured as “state anxiety” using the State-Trait Anxiety Inventory (STAI)-Form JYZ.

### Liveliness, depression, and boredom

Liveliness consisted of emotions such as feeling “fresh” (the pleasant state of feeling cheerful and the good state of feeling refreshed), depression consisted of emotions such as anxiety (being in a state of having concerns about what will happen and not being able to shake such a feeling), and Boredom consisted of sensations such as “being tired” (exhaustion of physical and mental strength, leading to decline in such functions). A relaxed state is represented by higher scores for liveliness and lower scores for fatigue and depression.

### New Japanese version of STAI

The new Japanese version of STAI is a psychological test used as a scale to measure anxiety. It was standardized for Japanese people on the basis of STAI (form Y) that was originally published by C.D. Spielberger.^5^ The state anxiety scale assesses how respondents feel at the moment. Trait anxiety assesses relatively stable reaction tendencies toward uneasy experiences, such as how an individual generally always feels (i.e., it assesses personality traits prone to anxiety).^6^

### Analysis methods

The sample included 24 individuals for analysis. However, only 23 individuals in whom the HF and LF/HF domains could be analyzed and who provided responses for STAT trait anxiety were included in the analyses. Participant attributes obtained by using self-administered questionnaire were used to calculate basic statistics. Physiologic indices were compared between the groups using the Friedman test. Measurement items that showed a significant difference underwent multiple comparisons using a Wilcoxon signed-rank test, and corrections were performed using Bonferroni inequality. The two groups were compared to find variation 1 (immediately after intervention − before intervention), variation 2 (15 minutes after intervention − before intervention), and variation 3 (15 minutes after intervention − immediately after intervention) using a Wilcoxon signed-rank test. Psychological indices were compared within the groups using a Wilcoxon signed-rank test. A comparison between the two groups was performed using a Wilcoxon signed-rank test to find variation 2 (15 minutes after intervention – before intervention). Analyses were performed using the statistical software SPSS Statistics 20 (IBM, Armonk, NY), and a *p*-value <0.05 was considered to represent a statistically significant difference.

### Ethical considerations

This study was performed with the approval of the ethics committee of the affiliated institution (approval number C-1011). Written consent was obtained after the study purpose and content were explained to the participants.

## Results

### Environmental and subject attributes

The study was performed in the training room at K University, where the temperature was maintained at 23.9°C ± 0.73°C (mean ± standard deviation, hereafter) and humidity was maintained at 61.9% ± 1.09%. The study sample consisted of 24 female students with a mean age of 21.2 ± 0.59 years. The participants' average height, weight, and body–mass index were 157.5 ± 4.73 cm, 49.9 ± 4.60 kg, and 20.1 ± 1.83 kg/m^2^, respectively. The participants' STAI trait anxiety scores based on STAI standard evaluation stage were normal (stage 3) in 56.5%, high (stage 4) in 17.4%, very high (stage 5) in 17.4%, and low (stage 2) in 8.7% of the participants. The mean score was 44.7 ± 8.83 points, which was classified as stage 3 (normal) (Tables 3 and 4).

**Table 3.** Participants Characteristics (*n* = 24)

*Characteristic*	*Minimum*	*Maximum*	*Mean* ± SD
Age (yr)	20	23	21.2 ± 0.59
Height (cm)	147	165	157.5 ± 4.73
Weight (kg)	41	57	49.9 ± 4.60
Body–mass index (kg/m^2^)	17	23	20.1 ± 1.83
Trait anxiety (points)	32	61	44.7 ± 8.83

SD, standard deviation.

**Table 4.** STAI Standard Score Stage Classification

*Stage*	*Trait anxiety score*	*Patients (*n* = 23) (%)*
I: Very low	21–23	0.0
II: Low	24–33	8.7
III: Normal	34–44	56.5
IV: High	45–54	17.4
V: Very high	55–80	17.4

### Changes in physiological indices

#### Blood pressure

The changes in physiologic indices are shown in Tables 5 and 6. Systolic blood pressure did not significantly differ between the two groups or within the groups. Variation 3 in diastolic pressure was 2.5 ± 5.47 mmHg in the intervention group and −0.6 ± 5.92 mmHg in the control group (z = −2.104; *p* < 0.05). No statistically significant differences were noted within the groups.

**Table 5.** Intragroup Comparison of Changes in Physiologic Indices over Time

					p*-Value*			
*Item*	*Group*	*Before intervention*	*Immediately after intervention*	*5 min after intervention*	*Comparison 1*^a^	*Comparison 2*^b^	*Comparison 3*^c^	*Comparison 4*^d^
SBP (mmHg) (*n* = 24)	Intervention group	101.0 ± 9.05	97.3 ± 6.70	100.1 ± 9.53	NS	–	–	–
	Control group	100.2 ± 11.54	97.0 ± 10.05	97.0 ± 7.83	NS	–	–	–
DBP (mmHg) (*n* = 24)	Intervention group	58.9 ± 7.30	57.1 ± 5.80	59.5 ± 6.09	NS	–	–	–
	Control group	58.4 ± 10.06	58.0 ± 8.48	57.5 ± 6.74	NS	–	–	–
HF (msec^2^/Hz) (*n* = 23)	Intervention group	530.0 ± 533.6	830.0 ± 988.5	883.0 ± 801.2	<0.01	NS	NS	NS
	Control group	771.0 ± 712.3	713.0 ± 787.2	806.0 ± 787.6	NS	–	–	–
LF/HF (*n* = 23)	Intervention group	2.2 ± 1.63	1.9 ± 1.50	2.2 ± 2.07	NS	–	–	–
	Control group	2.0 ± 1.71	1.8 ± 1.67	2.1 ± 1.57	NS	–	–	–

Unless otherwise noted, values are the mean ± SD.

^a^ Before intervention vs. immediately after intervention vs. 15 minutes after intervention: Friedman test.

^b^ Before intervention vs. immediately after intervention: Wilcoxon signed-rank test (Bonferroni corrected).

^c^ Before intervention vs. 15 minutes after intervention: Wilcoxon signed-rank test (Bonferroni corrected).

^d^ Immediately after intervention vs. 15 minutes after intervention: Wilcoxon signed-rank test (Bonferroni corrected).

NS, not significant; –, did not examine Wilcoxon signed-rank test because significant difference was not provided by Friedman test.

**Table 6.** Comparison Between the Two Study Groups for Variation in Physiologic Indices

*Variable*	*Group*	*Variation 1*^a:^*intervention − before intervention*	p*-Value*	*Variation 2*^b:^*15 min after intervention − before intervention*	p*-Value*	*Variation 3*^c^*: 15 min after intervention − immediately after intervention*	p*-Value*
SBP (mmHg) (*n* = 24)	Intervention group	−3.8 ± 7.72	NS	−0.9 ± 9.45	NS	2.9 ± 7.73	NS
	Control group	−3.2 ± 8.58		−3.2 ± 8.18		0.0 ± 5.75	
DBP (mmHg) (*n* = 24)	Intervention group	−1.8 ± 6.50	NS	0.6 ± 6.99	NS	2.5 ± 5.47	<0.05
	Control group	−0.3 ± 7.18		−0.9 ± 6.49		−0.6 ± 5.92	
HF (msec^2^/Hz) (*n* = 23)	Intervention group	300 ± 552.2	<0.01	353 ± 602.8	NS	52 ± 659.4	NS
	Control group	−58 ± 681.3		35 ± 769.0		93 ± 430.0	
LF/HF (*n* = 23)	Intervention group	−0.3 ± 1.64	NS	0.0 ± 1.88	NS	0.3 ± 1.81	NS
	Control group	−0.2 ± 1.35		0.1 ± 1.49		0.3 ± 0.91	

Unless otherwise noted, values are the mean ± SD.

^a^ Intervention group vs. control group for variation 1: Wilcoxon signed-rank test.

^b^ Intervention group vs. control group for variation 2: Wilcoxon signed-rank test.

^c^ Intervention group vs. control group for variation 3: Wilcoxon signed-rank test.

#### Indices of heart rate variability

Variation 1 in HF domain was 300 ± 552.2 msec^2^/Hz in the intervention group and 58 ± 681.3 msec^2^/Hz in the control group. A significant increase was observed in the intervention group (z = −2.981; *p* < 0.01). The mean HF domain in the intervention group was 530 ± 533.6 msec^2^/Hz before the intervention, 830 ± 988.5 msec^2^/Hz immediately after the intervention, and 883 ± 801.2 msec^2^/Hz 15 minutes after the intervention, showing a significant difference (*p* < 0.01). LF/HF domain did not significantly differ between or within the groups.

### Changes in psychological indices

#### Liveliness (“fresh”), depression (“anxious”), and boredom (“tired”)

The changes in psychological indices are shown in Tables 7 and 8. For liveliness, variation 2 was 29.1 ± 24.31 points in the intervention group and 13.3 ± 23.34 points in the control group, with a significant increase observed in the intervention group (z = −2.959; *p* < 0.01). The mean scores in the intervention group were 43.1 ± 17.99 points before the intervention and 72.2 ± 16.63 points 15 minutes after the intervention, showing a significant difference (z = −4.001; *p* < 0.01). In the control group, the mean scores were 44.4 ± 23.24 points before the intervention and 57.7 ± 20.07 points 15 minutes after the intervention, showing a significant difference (z = −2.267; *p* < 0.05).

**Table 7.** Intragroup Comparison of Changes in Psychological Indices over Time (*n* = 24)

*Item (score)*	*Group*	*Before intervention*	*15 min after intervention*	p*-Value*^a^
Liveliness (“fresh”)	Intervention group	43.1 ± 17.99	72.2 ± 16.63	<0.01
	Control group	44.4 ± 23.24	57.7 ± 20.07	<0.05
Depression (“anxious”)	Intervention group	32.8 ± 26.32	13.7 ± 21.43	<0.01
	Control group	37.7 ± 33.88	24.5 ± 32.05	<0.01
Boredom (“tired”)	Intervention group	35.9 ± 30.58	14.8 ± 18.67	<0.01
	Control group	37.3 ± 28.54	23.6 ± 20.42	<0.05
STAI (trait anxiety)	Intervention group	39.3 ± 5.95	31.1 ± 5.57	<0.01
	Control group	40.8 ± 9.26	36.7 ± 8.23	<0.01

Unless otherwise noted, values are the mean ± SD.

^a^ Before intervention vs. 15 minutes immediately after intervention: Wilcoxon signed-rank test.

**Table 8.** Comparison Between the Two Study Groups for Variations in Psychological Indices

*Item (score)*	*Group*	*Variation 2: 15 min after intervention − before starting*	p-*Value*^**a**^
Liveliness (“fresh”)	Intervention group	29.1 ± 24.31	<0.01
	Control group	13.3 ± 23.34	
Depression (“anxious”)	Intervention group	−19.1 ± 20.25	NS
	Control group	−13.2 ± 19.44	
Boredom (“tired”)	Intervention group	−21.1 ± 30.58	NS
	Control group	−13.6 ± 27.38	
STAI (trait anxiety)	Intervention group	−8.2 ± 6.92	<0.01
	Control group	−4.1 ± 5.78	

Unless otherwise noted, values are the mean ± SD.

^a^ The intervention group vs. the control group for variation 2: Wilcoxon signed-rank test.

For depression, no significant difference was observed. The mean scores in the intervention group significantly decreased from 32.8 ± 26.32 points before the intervention to 13.7 ± 21.43 points 15 minutes after the intervention (z = −3.508; *p* < 0.01). In the control group, the mean scores significantly decreased from 37.7 ± 33.88 points before the intervention to 24.5 ± 32.05 points 15 minutes after the intervention (z = −2.939; *p* < 0.01).

For boredom, no significant difference was observed. The mean scores in the intervention group significantly decreased from 35.9 ± 30.58 points before the intervention to 14.8 ± 18.67 points 15 minutes after the intervention (z = −3.111; *p* < 0.01). The mean scores in the control group significantly decreased from 37.3 ± 28.54 points before the intervention to 23.6 ± 20.42 points 15 minutes after the intervention (z = −2.016; *p* < 0.05).

#### STAI state anxiety

Variation 2 was −8.2 ± 6.92 points in the intervention group and −4.1 ± 5.78 points in the control group, showing a significant decrease in the intervention group (z = −2.636; *p* < 0.01). The mean scores in the intervention group significantly decreased from 39.3 ± 5.95 points before the intervention to 31.1 ± 5.5 points 15 minutes after the intervention (z = −3.803; *p* < 0.01). The mean scores in the control group significantly decreased from 40.8 ± 9.26 points before the intervention to 36.7 ± 8.23 points 15 minutes after the intervention (z = −2.976; *p* < 0.01).

## Discussion

The present study was designed to clarify the physiologic and psychological effects of head treatment in the supine position using physiologic and psychological indices in female students. Physiologic effects included systolic blood pressure, diastolic blood pressure, HF domain, and LF/HF domain as indices of heart rate variability. Psychological indices included active pleasure, depression, and fatigue, which were measured using VAS, as well as anxiety as a scale of emotion, which was measured using STAI.

### Effects perceived from physiologic indices

Massage stimulation activates various receptors at sensory nerve endings. Signals are then sensed as they reach the cerebral cortex via the diencephalon through the spinal cord and are acknowledged as sensations of pressure, heat, and comfort. The signals sensed reach the central nervous system via afferent nerves and then the cerebral cortex via the diencephalon through the spinal cord and are acknowledged as massage sensations (pressure, heat, and pleasure sensations). In this stage, when the cerebral cortex is reached via the peripheral nervous system, it is believed that axonal and spinal reflexes of the nerve terminal are activated, and a complex reflex mechanism occurs in high-order neurons, which leads to the manifestation of the various effects of massage. Furthermore, massage works directly on blood vessels and lymph nodes, physically pushing stagnant blood and lymph fluid by applying pressure and then easing the pressure for blood and lymph to flow in. Repeating this action improves local circulation and also affects the nervous, vascular, and endocrine systems through various reflex mechanisms via the nerves. These interactions combine in a complex way and lead to the manifestation of the effects.^7,8^ In earlier studies on the effects of massage, it was reported that stress is eased and fatigue is reduced.^9^ It was also reported that massage reduces physical symptoms and provides relaxation.^10^

In the present study, HF was used as an index for parasympathetic nerve activity. When a head treatment was performed, differences were noted in HF measurements before the intervention, immediately after the intervention, and 15 minutes after the intervention. In addition, HF tended to increase the most immediately after the intervention compared with before the intervention. Furthermore, compared with bed rest only, values were greater immediately after the intervention compared with before the intervention. These findings suggest that performing a head treatment stimulated the parasympathetic nerves and increased their activity from before starting to immediately after the intervention.

When a head treatment was performed, diastolic blood pressure values were greater at 15 minutes after the intervention than those immediately after the intervention. This suggests that when a head treatment was performed, parasympathetic nerve activity at first increased immediately after the intervention and then gradually decreased over the next 15 minutes. The role of the autonomic nervous system is to maintain homeostasis within the body by regulating internal organ function. An individual organ basically has double innervation, with both sympathetic and parasympathetic nerves projecting into the organ. The autonomic nervous system has antagonistic innervation.^11,12^ Therefore, it is believed that the intervention group momentarily went into a state of accelerated parasympathetic nerve activity immediately after intervention; however, once head treatment was completed, the parasympathetic nerve predominant state tended to gradually ease.

In the present study, diastolic blood pressure and LF/HF were used as physiologic indices. They both decreased immediately after the intervention (increased parasympathetic nerve activity) and showed increasing tendencies over the first 15 minutes after the intervention (parasympathetic nerve activity temporarily increased before gradually decreasing).

### Effects perceived from psychological indices

When a head treatment was performed, the feeling of liveliness increased, depression and boredom decreased, and the state of anxiety decreased. In particular, performing a head treatment increased the feeling of liveliness and decreased the state of anxiety more than did bed rest alone. “Refreshment” represented the comfortable state of feeling cheerful and the good state of feeling refreshed. It was classified in the emotional factor of liveliness. A state of anxiety signifies a temporary state caused by psychological stress or conflict associated with interpersonal relationships. Common specific factors include stress, interpersonal relationships, and anxiety regarding the future. A state of anxiety can be improved if these factors can be removed or eased.

In the present study, performing a head treatment not only decreased depression and fatigue but also markedly increased liveliness and reduced anxiety. These findings suggest that a head treatment is a massage technique that offers relaxing and refreshing effects. With further verification in the future, this technique could be useful in easing anxiety and depressive symptoms.

### Study limitations and future tasks

Because the present study included only healthy female students, the sample was limited. Furthermore, the time slot for the head treatment was set as per the participants' request. Consequently, the effects may have been considerably influenced by the circadian rhythm. In future studies, the same time slot should be used for both the intervention and control groups. Moreover, because the menstrual cycle of young healthy women affects their autonomic nerves and body temperature regulation, the low-temperature phase should be included as a condition for participation. In future, effects should be examined in a population with a broad age range and upon continued implementation of head treatment. Thus, a future task will be to establish head treatment as a safe and effective treatment that can be combined with pharmacotherapy for patients with diseases who have a chief complaint of anxiety and depression.

## Conclusions

This study was conducted with a crossover design in which the same 24 healthy female students were included in the intervention group (head treatment) and the control group (bed rest alone). With intervals of at least 1 week between each treatment, the physical and psychological effects of a head treatment were examined, and the investigation revealed the following.

First, the results suggested that when a head treatment was performed, parasympathetic nerve activity increased immediately after the intervention and gradually decreased thereafter. The results demonstrated that a head treatment is a massage technique that can be safely performed without greatly affecting hemodynamics. Second, the results suggested that performing a head treatment not only decreased depression, boredom, and anxiety but also markedly increased liveliness. These findings demonstrated that a head treatment is a massage technique that offers relaxing and refreshing effects. Thus, on the basis of these results, it is suggested that head treatment has relaxing and refreshing effects and can be used as a technique to provide comfort.
